# Response Mechanisms of Woody Plants to High-Temperature Stress

**DOI:** 10.3390/plants12203643

**Published:** 2023-10-22

**Authors:** Chao Zhou, Shengjiang Wu, Chaochan Li, Wenxuan Quan, Anping Wang

**Affiliations:** 1 Key Laboratory for Information System of Mountainous Area and Protection of Ecological Environment of Guizhou Province, Guizhou Normal University, Guiyang 550001, China; chaozhou@gznu.edu.cn (C.Z.); chaochanl@gznu.edu.cn (C.L.); 2 Guizhou Academy of Tobacco Science, Guiyang 550081, China; wushengjiang1210@163.com

**Keywords:** woody plants, high-temperature stress, physiology and biochemistry, heat-resistant mechanism, genomics

## Abstract

High-temperature stress is the main environmental stress that restricts the growth and development of woody plants, and the growth and development of woody plants are affected by high-temperature stress. The influence of high temperature on woody plants varies with the degree and duration of the high temperature and the species of woody plants. Woody plants have the mechanism of adapting to high temperature, and the mechanism for activating tolerance in woody plants mainly counteracts the biochemical and physiological changes induced by stress by regulating osmotic adjustment substances, antioxidant enzyme activities and transcription control factors. Under high-temperature stress, woody plants ability to perceive high-temperature stimuli and initiate the appropriate physiological, biochemical and genomic changes is the key to determining the survival of woody plants. The gene expression induced by high-temperature stress also greatly improves tolerance. Changes in the morphological structure, physiology, biochemistry and genomics of woody plants are usually used as indicators of high-temperature tolerance. In this paper, the effects of high-temperature stress on seed germination, plant morphology and anatomical structure characteristics, physiological and biochemical indicators, genomics and other aspects of woody plants are reviewed, which provides a reference for the study of the heat-tolerance mechanism of woody plants.

## 1. Introduction

Woody plants are important components of ecosystems, usually perennial, with complex root and stem structures, and play an important role in limiting carbon dioxide (CO_2_) and other greenhouse gases [1]. Woody plants are also the main biomass resources of biofuels [2]. Because woody plants are perennial and have a long life cycle, compared to herbs, they experience extreme abiotic stress in their lives [3]. In recent years, due to the continuous emission of greenhouse gases, the global temperature has increased year by year. With the intensification of global climate change, high temperature has become one of the important environmental factors restricting the growth and development of woody plants. Each plant species naturally exhibits its own optimal temperature range for growth and reproduction. When this temperature is higher than the optimal temperature and reaches a threshold that is detrimental to the growth or survival of the species, we classify this temperature as high temperature [4]. Of course, different species have different tolerances to temperature, so the range of high temperatures is also different. At present, most of the effects of high-temperature stress on plants are aimed at the Gramineae family, such as *Arabidopsis thaliana* and crop species, and the research on woody plants is usually limited. Therefore, how woody plants respond to the extreme environment of global warming and their response to the climate are our primary concerns [5]. Because of the sensitivity of woody plants to environmental changes, it is of great significance to study their physiological changes and ecological characteristics in adapting to high-temperature environments to promote sustainable development and ecological environment protection [6]. In this paper, the effects of high-temperature stress on woody plants were systematically reviewed from the aspects of seed germination, plant morphology and anatomical structure, physiological and biochemical indicators, genomics and so on.

## 2. Effect of High-Temperature Stress on the Seed Germination of Woody Plants

The seed germination stage is sensitive to the external environment; there are many factors that affect the seed germination stage, and temperature is one of the main environmental factors that determine seed germination [7]. When the temperature exceeds a certain limit, the water absorption and oxygen intake of seeds is limited [8], significantly affecting germination time, slowing the germination rate and reducing the germination potential [9]. This may be because high temperature strengthens seed dormancy, thus, affecting seed germination. Some woody plant seeds have dormancy mechanisms to ensure that seeds germinate under suitable environmental conditions; high-temperature stress may interrupt or change the dormancy state of seeds, resulting in the abnormal germination of seeds [10]. In addition, high-temperature stress can also lead to lipid peroxidation and protein inactivation and affect the enzyme activity of seeds, thus, increasing the seed mortality [11]. To avoid the negative impact of high-temperature stress on the seed germination and seedling growth of woody plants, some measures can be taken, such as maintaining appropriate temperature and humidity conditions at the seed germination stage, providing sufficient water and nutrients and avoiding exposure to a high-temperature environment.

## 3. Effects of High-Temperature Stress on the Morphological and Anatomical Characteristics of Woody Plants

### 3.1. Effects on Woody Plant Morphology

Plant morphology refers to the characteristics of plants in terms of morphological structure, which can reflect the growth and physiological state of plants and be used to better understand and study the growth and development process and ecological characteristics of plants [12]. Leaves are the main organs of photosynthesis and respiration in plants, which are extremely sensitive to high temperature, and their growth performance is directly damaged by heat stress [13]. Its morphological characteristics are an important reference index for the study of stress resistance in woody plants [14]. Under high-temperature stress, leaves curl, brown and shrink [15]. The growth rate of the trunk and crown slows down, and long-term stress leads to short plant morphology, thin branches and decreased growth ability [16]. Bark cracks and dries, leading to the loss of nutrients and water, and the strength and stability of the stem are reduced [17]. The accelerated depletion of plant water under high temperatures is usually accompanied by water stress [18]. Plants normally lower their body temperature through transpiration but may reduce transpiration to conserve water under conditions of insufficient water supply, resulting in dehydration and impaired growth [19]. However, plants activate specific gene expression and signaling pathways when responding to high-temperature and water stress. Sometimes these signaling pathways overlap; that is, there is an interaction between the high-temperature signaling pathway and the water signaling pathway, causing the plant to respond jointly to these two stresses and jointly resist the adverse effects of the plant [20]. The main reason for these phenomena is that high-temperature stress accompanied by water stress leads to large amounts of water evaporation, plant damage and reduced enzyme activity. Under high temperature stress, woody plants change their morphology with temperature changes to protect themselves. It also shows that high-temperature damages the morphology of woody plants, and a long-term high-temperature environment adversely affects the growth and development of woody plants.

### 3.2. Effects on Anatomical Structure Characteristics of Woody Plants

The anatomical features of leaves, trunks and roots in woody plants have been reported, but most of them were studied in leaves, which may be related to the sensitivity of the anatomical features of leaves to abiotic factors [21]. Therefore, the anatomical characteristics of leaves are the main characteristics used to explore the adaptive response of plants to environmental conditions [22]. The anatomical structure characteristics of woody plants under high-temperature stress are shown in Table 1. The anatomical structure of plants is closely related to their growth environment, and different growth environments have different effects on the anatomical structure of plants, thus, affecting the growth and survival of plants [23]. Under the stress of a high temperature, woody plants respond in morphology and internal structure and then adapt to the high-temperature environment to protect the body from harm.

## 4. Effects of High-Temperature Stress on Physiological and Biochemical Indexes of Woody Plants

**Figure 1:**
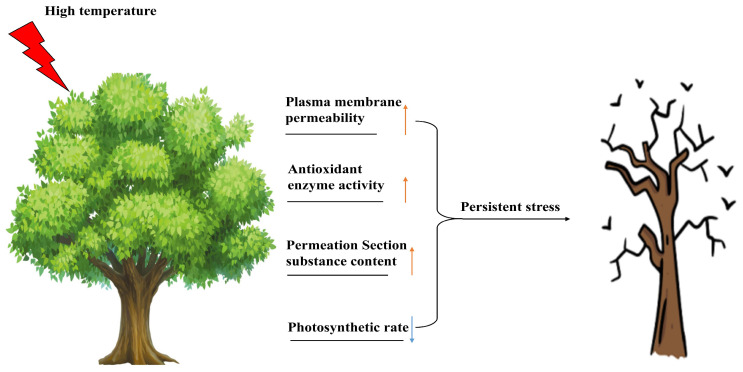
Effects of high-temperature stress on physiology and biochemistry of woody plants. Upward-pointing arrows indicate activated/upregulated physiological indices. Downward-pointing arrows indicate deactivated/downregulated physiological indices.

In most cases, some common effects of high temperature on the physiological and biochemical reactions of woody plants are shown in Figure 1. Many physiological and biochemical indicators, such as membrane thermal stability, antioxidant enzyme activity, osmotic regulatory substances and photosynthetic characteristics, are affected. Table 2 shows the similarities and differences in response mechanisms and molecular mechanisms of different species under high-temperature stress.

### 4.1. Influence on Plasma Membrane Permeability

The plasma membrane (PM) forms a physical barrier to close the intracellular compartment and mediate direct communication between plants and the environment. The permeability of the plasma membrane is very important for maintaining plant development and plant–environment interactions [38]. Studies have shown that high temperatures increase the content of malondialdehyde and reactive oxygen species in woody plants, as well as the peroxidation of membrane lipids, leading to an increase in the permeability of the plasma membrane, as well as the increased permeability of cell membranes to ions, small molecules, and water. The ion balance is disrupted, thereby affecting the structure and function of the plasma membrane [39]. Malondialdehyde (MDA) is the final decomposition product of membrane lipid peroxidation, and the MDA content in leaves effectively reflects the degree of membrane lipid peroxidation under high-temperature stress [40]. There are reactive oxygen species (ROS) in woody plants at high temperature, such as singlet oxygen (^1^O_2_), superoxide radical (O_2_^−^), hydrogen peroxide (H_2_O_2_) and hydroxyl radical (OH^−^) [41]. It can easily lead to oxidative stress and damage to the lipid membrane [42]. The study found that as the stress temperature increased, the contents of MDA and H_2_O_2_ increased, and carotenoids also accumulated in large quantities. High-temperature stress changes gene expression in the carotenoid synthesis pathway, and the accumulation of carotenoids increases antioxidant activity and provides a protective mechanism [43]. Similar reports were reported in *Canarium album* [44], *Citrus reticulata* Blanco [45], and mangrove forests [46]. As shown in literature, the PM and endoplasmic reticulum (ER) participate in high-temperature stress reactions in plants, which induce H_2_O_2_ production and the ROS redox signaling pathway. Changes in the physical state of the membrane affect the membrane proteins and membrane structure, resulting in changes in PM permeability, which then affect a series of cell reactions [47]. The body mitigates adverse effects through adaptive mechanisms, mainly including antioxidant defense, synthesis of thermostable proteins and membrane lipid regulation [48]. Plants synthesize antioxidant enzymes and substances to reduce oxidative stress, help protect plasma membrane lipids and proteins from oxidative damage and slow the increase in permeability [49]. Plants synthesize thermoproteins, which help maintain the stability of plasma membrane proteins and prevent the denaturation and inactivation of other proteins [50]. Plants can synthesize lipids with greater thermal stability to maintain plasma membrane integrity and fluidity. In addition to this, osmoregulatory substances interact with plasma membrane permeability. The accumulation of osmoregulatory substances helps maintain the stability of the plasma membrane and mitigate adverse effects on cells. Changing the permeability of the plasma membrane affects intracellular material transport and signal transmission, adversely affecting the normal growth of woody plants [51].

### 4.2. Influence on Osmotic Adjustment Substances

Woody plants can accumulate some organic or inorganic substances independently when they are stressed by high-temperature adversity [52]. The main organic substances involved in osmotic adjustment are soluble sugar, proline (Pro) and soluble protein [53]. Studies have found that high-temperature stress can cause an imbalance of water inside and outside the cells of woody plants, leading to cell dehydration. The accumulation of soluble sugar increases the osmotic concentration of cells and attracts water into cells, thereby alleviating dehydration symptoms. Sugar synthesis and accumulation can also provide plants with additional carbon sources and energy reserves, helping to regulate cell permeability [54]. For example, high temperature increases the contents of glucose and fructose in *Cunninghamia lanceolata* and *Pinus ponderosa*, providing a carbon source and energy to cope with stress, thus, increasing their heat resistance [55]. Moreover, sugar, as a signal molecule, plays a key role in regulating plant development [56,57]. High temperature induces an increase in the activity of enzymes related to proline synthesis and promotes the accumulation of proline. The accumulation of proline helps maintain the osmotic balance of cells, slow cell dehydration, and maintain cell morphology and function, thereby improving plant adaptability to high temperatures [58]. For example, in *Bruguiera gymnorrhiza* [59] and *Ginkgo biloba* [60] under high-temperature stress, the proline content increased obviously, and the activities of metabolic enzymes such as proline synthase were also improved. Proline is also involved in regulating the redox balance of plants, inhibiting the accumulation of ROS and improving the heat resistance of plants [61]. High temperature increases the content of soluble proteins in plant cells, especially inducing the expression of thermal protein genes and increasing the synthesis of thermal proteins. These thermoproteins are transcribed and translated into proteins, which then help other proteins remain stable against damage from osmotic stress [62]. Other studies have found that high-temperature stress can lead to the degradation and damage of soluble proteins in woody plants and reduce the content of soluble proteins [54]. Some people think that different species have different substances that mainly rely on regulating cell osmotic potential, and different varieties and stress treatments have an impact on the changes in the osmotic adjustment factors of woody plants [63]. Under high-temperature stress, woody plants induce the accumulation of osmotic regulatory substances such as soluble sugar, Pro, and soluble protein in the body, thereby regulating the osmotic pressure of the internal environment. Osmoregulation also retains water and slows water loss by reducing transpiration. Accelerating cell wall synthesis increases cell stability, interacts with the accumulation of osmotic regulatory substances, and maintains the osmotic balance and stability of the cell structure [64].

### 4.3. Effect of Antioxidase Activity

**Figure 2:**
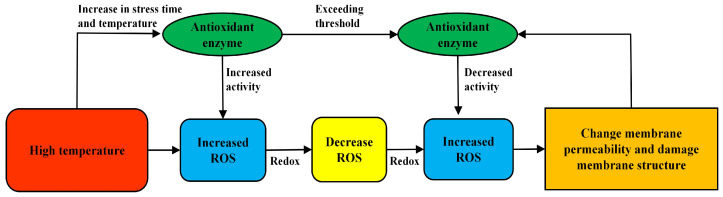
Effect of high-temperature stress on antioxidant enzyme activity of woody plants.

Reactive oxygen species (ROS) in plants are mainly eliminated by antioxidant enzymes. The antioxidant enzymes in woody plants mainly include catalase (CAT), superoxide dismutase (SOD), glutathione peroxidase (GPX), peroxidase (POD) and ascorbate peroxidase (APX) [65]. The antioxidant defense mechanism of enzymes is very important for plants in stress-resistant environments. If antioxidant enzymes are affected, ROS accumulate excessively, which has adverse effects on plant growth and resistance to adversity [66]. High-temperature stress can increase the activities of antioxidant enzymes, such as SOD, APX and CAT, which increase with increasing temperature, but their activities tend to decrease when the temperature exceeds a certain limit. It is considered that the temperature has exceeded the tolerance threshold, which leads to structural damage and decreases the activity of antioxidant enzymes [67]. In *Rhododendron* [68], *Cedrus deodara* [31] and *Ficus altissima* [35], there are similar reports in other woody plants. Increased antioxidant enzyme activity helps protect woody-plant-cell structures and biochemical molecules from oxidative stress damage. They also interact with other physiological mechanisms, such as osmoregulation. Some soluble proteins have antioxidant activity. It can neutralize reactive oxygen species generated by oxidative stress and adjust the intracellular redox balance [69], as well as reduce oxidative damage to cell membranes and intracellular organelle structures. Together, they maintain the stable growth and survival of woody plants under high-temperature conditions [70]. This shows that woody plants can improve the activity of antioxidant enzymes, reduce the oxidation of cells and maintain structural stability under high-temperature stress. However, antioxidant enzymes can only play a limited defensive role. When the stress pressure of woody plants exceeds the threshold, adversity inhibits the expression of their enzymes, reduces the activity of antioxidant enzymes, accumulates oxidized substances in plants and, finally, destroys the cell structure (Figure 2).

### 4.4. Effects on Photosynthetic Characteristics

**Figure 3:**
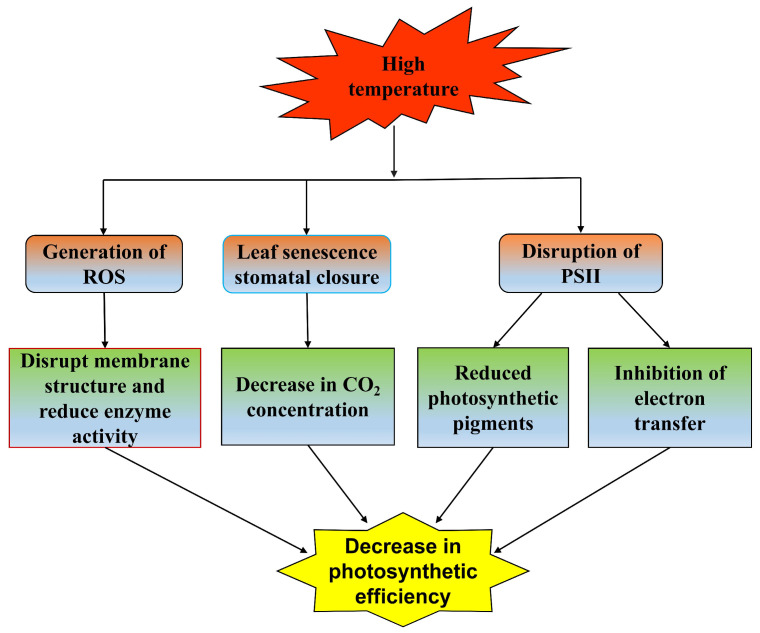
Effects of high-temperature stress on photosynthetic characteristics.

Photosynthesis is very sensitive to temperature, and small temperature fluctuations in a short time also have an impact on photosynthesis [71]. High-temperature stress directly affects CO_2_ assimilation, photochemical reactions and the synthesis of photosynthetic pigments [72]. The change in chlorophyll content can reflect the stress degree of plants [73]. The results show that high-temperature stress reduces the contents of chlorophyll a and chlorophyll b in woody plants, reducing the photosynthetic rate. There are differences in carotenoid content in different tree species [39]. Carotenoids have a strong antioxidant capacity and can reduce damage to plants under high-temperature stress [74]. High temperature triggers oxidative stress, resulting in excessive ROS production, which damages the photosynthetic complex and chloroplast membrane and thylakoid membrane and reduces the activity of enzymes, such as RuBisCo-activated enzyme, whose activity is affected by the precise spatial conformation of its components [75]. High temperature leads to the structural change in RuBisCo, which makes its conformation disordered and causes it to lose its ability to combine with CO_2_. It also reduces the activities of photosystem II (PSII) and ATPase, which further affects the efficiency of photosynthesis [76]. High temperature causes leaf senescence and water loss, and a large number of stomata close, which further reduces the intracellular CO_2_ concentration and water evaporation, leading to a decrease in the photosynthetic rate. The change in chlorophyll fluorescence parameters can describe the change in photosynthesis, thus, evaluating the response and adaptability of plants to high temperature [77]. The chlorophyll fluorescence quantum yield (Fv/Fm) of plants decreased, indicating that the photochemical efficiency of the PSII reaction center decreased, which may be caused by incomplete assembly of the photosynthetic pigment complex and serious damage to the structure and function of the reaction center [78]. It also led to a decrease in the maximum fluorescence value (Fm), indicating that the PSII reaction center was destroyed. The electron transfer of photosynthesis was inhibited, and the actual fluorescence value (Ft) decreased [79]. In addition to the above indicators, it also leads to the upregulation of chlorophyll fluorescence nonphotochemical quenching (NPQ) [80]. The efficiency of photosynthesis under high-temperature stress is also related to the antioxidant system and osmotic regulation. High temperature stimulates woody plants to increase the activity of antioxidant enzymes. Moreover, it helps remove ROS, reduce the impact of oxidative stress, and protect the integrity of photosynthetic complexes and chloroplast membranes, thereby maintaining the normal progress of photosynthesis [67]. Similarly, high temperatures cause the accumulation of soluble proteins, enhance resistance to oxidative stress, reduce ROS damage to chloroplasts, and coordinate to respond to high temperature stress [69]. High-temperature stress affects the photosynthetic characteristics of woody plants by affecting the production of light and pigments, decomposing the generated light and pigments, damaging the photosynthetic reaction center structure and reducing the activities of enzymes involved in photosynthesis (Figure 3).

## 5. Effects of High-Temperature Stress on the Genomics of Woody Plants

High-temperature stress affects the genomics of woody plants, including gene expression regulation, signal transduction pathways, epigenetic regulation, genome variation and mutation, and the regulation of heat shock proteins, which leads to changes in the physiological and biochemical processes of plants [30]. High-temperature stress affects the expression of transcription factors in woody plants. By regulating the expression level of stress-responsive genes, alone or together with other transcription factors, the transcription and translation of genes is affected to improve the heat stress tolerance of plants [81]. The genome-wide expression profile of heat shock transcription factors (*HSFs*) has been studied in various species, and their structure and function are complex and diverse; *HSFs* are also the most important regulatory transcription factors of woody plants under high-temperature stress [82,83]. After woody plants were subjected to high-temperature stress, *HSFs* were induced significantly and accumulated continuously, thus, activating the expression of heat shock proteins (*HSPs*), *APX2* and other genes and enhancing heat tolerance. It also participates in various physiological processes by regulating target genes related to growth and development, metabolism and abiotic stress and plays a role in maintaining cell homeostasis [84]. At the same time, *HSFs* are upregulated by *DREB* (stress response element binding protein) [85]. The expression of *HSFA3*, *HSFA4*, *HSFA9*, *HSP90*, SOD and CAT can be induced to improve the heat tolerance of plants [86]. In addition to *HSFs*, other transcription factors, such as *bHLH*, *MYB*, *WRKY* and *NAC*, also play an important role in the response of plants to high temperature [87], as shown in Table 3.

*HSPs* are important molecular chaperones that promote other proteins to refold, stabilize and assemble after being induced by high-temperature stress. At the same time, the expression of many stress-induced genes is upregulated, such as molecular chaperones, active oxygen scavenging compounds, enzymes related to antioxidant metabolic reactions and osmotic adjustment substance synthesis [100]. Under high-temperature stress, plant HSFs can induce the expression of heat shock proteins (*HSPs*) (*sHSP/HSP20*, *HSP60*, *HSP70*, *HSP90*, *HSP100*), and five different HSPs cooperate to activate the transcription of HSFs and activate high-temperature signal transduction. At the same time, as molecular chaperones, they can assist the correct folding of proteins alone or together, thus, alleviating the damage caused by high temperature to woody plants [34]. To explore the evolutionary relationship and conserved domains of members of the heat-stress gene family in woody plants and to construct gene silencing vectors for agricultural crops will lay a foundation for studying the cultivation of high-temperature-resistant agricultural varieties and help to increase the yield of agricultural cash crops.

Epigenetic mechanisms such as DNA methylation, histone modification, histone variation and miRNA can affect gene expression and stability and reduce the damage caused by high temperature to plants [101]. DNA methylation involves the addition of a methyl group (CH3) to the cytosine position of DNA to form 5-methylcytosine, forming CG, CHG, and CHH (H stands for A, T, or C). It is an epigenetic change and reversible. The main mechanism of epigenetic modification; plant phenotypic changes induced under stress can respond to abiotic stress [102]. This process is widely used to cope with heat stress [103]. The results show that in most cases, after exposure to high temperature, the overall methylation level of plants is lower than that of control plants [104]. For example, at 25 °C, the methylation rate of poplar is 38.93%, while at 42 °C, the methylation rate is 28.61% [105]. In *Zea mays* L., CG and CHG, under high temperature stress, the hypomethylation of genomic DNA occurs in the environment. In *Glycine max*, DNA hypomethylation occurs at CHG and CHH sites in root hairs after heat treatment [106]. In *Brassica napus* cv., chicks are cultured with heat-stress treatment compared with controls. Spores for 6 h result in genome-wide DNA hypomethylation, especially in CG and CHG environments [107]. In *Arabidopsis thaliana*, DNA methylation during *Arabidopsis thaliana* seed development is moderately affected under mild heat stress; however, severe heat stress causes DNA methylation in the promoters and the genetic regions of germination-related genes. Significant changes in basalization occurred [108]. The study found that heat stress induces DNA demethylation in *Arabidopsis thaliana* genes but not in intergenic regions [103]. High-temperature stress reduces methylation levels, mainly due to the inhibition of DNA methylase activity, resulting in reduced DNA methylation levels [109]. In addition, it may also increase the activity of DNA demethylases. DNA demethylases are enzymes responsible for removing methyl groups from DNA molecules [110]. Therefore, high temperatures may lead to reduced levels of DNA methylation, in part due to increased demethylation of the methyl groups. Histone methylation and acetylation have not been well characterized in woody plants under heat stress. In other species, in *Arabidopsis thaliana*, histone methylation occurs mainly at Lys4 (K9), Lys9 (K27), Lys27 (K36), Lys36 (K17), Arg17 (R3) and Arg3 of histone H3 [111]. Compared with MLT stress, H3K9ac and H3K4me3 levels are higher under HT, which may lead to chromatin relaxation and thereby activate gene expression [112]. When grapes are exposed to a high temperature of 45 °C, the phosphorylation sites and acetyl sites undergo significant changes. Compared with phosphorylation, acetylation regulates more photosynthesis-related proteins and is more sensitive to high temperature. Acetylation can balance phosphorylation in terms of protein activity, and phosphorylation and acetylated proteins work together to affect the heat tolerance of grapes [113]. Studying epigenetics can help plants cope with high temperature stress, maintain cell homeostasis, and improve their adaptability. Although the research has made some progress, further research is still needed to understand the epigenetic mechanism in different plant species and under different high-temperature stress conditions to reveal its detailed regulatory network and mechanism of action. These studies are of great significance for improving the high-temperature tolerance of plants and the sustainability of plant production.

## 6. Summary and Prospect

The effects of high-temperature stress on woody plants were reviewed from the aspects of seed germination, plant morphology, physiology, biochemistry and genomics. Under high-temperature stress, the germination rate of plant seeds decreased, and the germination time was prolonged. Plant leaves are wrinkled and dry, and they fall off. Physiologically and biochemically, high temperature causes MDA and ROS accumulation and membrane lipid peroxidation, which leads to the destruction of cell membrane structure and function. Osmotic regulatory substances accumulate in the body to regulate cell osmotic pressure, maintain osmotic balance and stabilize cell structure. The activity of antioxidant enzymes is increased to reduce the oxidation of cells and maintain the stability of the structure. This will reduce the synthesis of photosynthetic pigments, destroy the cell structure and reduce the activity of enzymes, which will reduce photosynthetic efficiency. In genomics, changes in gene expression, DNA structure, protein synthesis and function further affect the growth and development of woody plants.

At present, many research achievements have been made on the effects of high-temperature stress on woody plants, but research on the heat resistance of woody plants has not yet formed a complete system. The future research should focus on the adaptation mechanism and regulation mechanism of woody plants under high-temperature stress and improve the tolerance of woody plants to high-temperature stress through gene editing. At the same time, we should also explore the different responses of different organs to high-temperature stress and different woody plants to high-temperature stress. Under the background of climate change, studying the genes related to the heat tolerance of woody plants is helpful to screen stress-resistant plant varieties and improve the stress-resistant ability of plants, which will significantly improve their yield and quality when used in agricultural crops. Exploring and expounding the physiological and molecular mechanisms of woody plants under high-temperature stress will also help to effectively select new varieties of woody plants with high-temperature resistance.

## Figures and Tables

**Table 1:** Effects of high-temperature stress on the anatomical structure characteristics of woody plants.

Anatomical Site	Change	Mechanism	References
Leaves	Increase: leaf thickness, cell wall thickness, stomatal density Decrease: mesophyll cell volume	Reduce water evaporation and heating area, improve water utilization efficiency	[24,25]
Stems	Increase: wall thickness, lignincontent Decrease: wood fiber diameter, length	Smaller hydraulic conductivity, reducing water loss; enhance protection and heat dissipation capabilities	[26,27]
Roots	Increase: Number and length of root hairs Decrease: Cell division	Increase absorption and utilization of water and nutrients	[28,29]

**Table 2:** Similarities and differences in response mechanisms and molecular mechanisms of different species under high-temperature stress.

Category	Similarities	Differences	References
Response mechanism	Activating gene expression: Changes in gene expression occur when plants are stressed Accumulation of antioxidant substances: Stress produces oxidative substances to neutralize them and reduce oxidative damage Regulate water balance: Control water evaporation by closing or opening stomata to maintain water balance Accumulation of resistance substances: Synthesis of resistance substances, such as proteins and lipids, in response to stress. Stomatal regulation: Under high temperature conditions, plants often close their stomata to reduce water transpiration	Ecological adaptability: The high temperature stress response mechanism of plants is affected by their ecological environment. Plants may have different high temperature adaptation strategies in different ecological environments Differences in hormone regulation: Different species may regulate different types of plant hormones under high temperature stress. For example, some species rely on abscisic acid, while others are more dependent on gibberellins.	[30,31,32,33]
Molecular mechanism	Synthesis of heat stress proteins: HSPs are usually synthesized in response to high temperature stress Antioxidant defense: High temperature stress triggers oxidative stress, which increases the activity of antioxidant enzymes Gene expression regulation: Respond to high-temperature stress by adjusting gene expression, including activation of heat-stress response genes	Differences in hormone regulation: Species regulate different types of phytohormones under heat stress Signaling pathways: Different plant species can activate different signaling pathways or specific stress response genes under high-temperature stress.	[34,35,36,37]

**Table 3:** Effects of high-temperature stress on transcription factors in woody plants.

Gene Family	Species	Gene Symbol	Response	Reference
HSF	*Camellia japonica*	*HSF-TEA*	Inducing gene expression, plants accumulate metabolites and activate different metabolic pathways and physiological and biochemical processes.	[88]
	*Eucalyptus robusta*	*EgHSF*	The expression levels of *EgHsf24* and *EgHsf32* genes increased significantly, thus, adapting to the high-temperature environment.	[89]
	*Juglans*	*JrHSF13*, *JrHSF22*	*JrHSF* promoted the accumulation of *HSP*, increased the denaturation temperature of protein and repaired the damaged protein to resist high temperature.	[90]
	*Citrus sinensis*	*CrHHSFB2*, *CrHSFB5*	*CrHsfB2* and *CrHsfB5* are important regulators of citric acid content, which changes during the degradation of citric acid caused by heat-stress.	[91]
	*Prunus salicina*	*PmHSF18*, *PmHSF2*	After heat shock, *PmHSF18* and *PmHSF2* became prominent HSF, and they participate in the key regulation of heat resistance.	[92]
	*Calyx glabrous*	*VHSF18*, *VHSF8*	The transcription levels of *VHSF18* and *VHSF8* increased sharply under high-temperature stress, which played a role in resisting heat resistance.	[93]
	*Ziziphus jujuba*	*ZjHSF-2*, *ZjHSF-3*	High temperature significantly upregulates the expression levels of *ZjHsf-2* and *ZjHsf-3* genes, which plays an important role in heat resistance.	[94]
	*Populus euphratica*	*PeHSFA2*	Poplar plants overexpressing *PeHSFA2* perform better than the control group under severe heat stress	[95]
bHLH	*Tamarix hispida*	*ThbHLH1*, *ThbHLH3*	The accumulation of *bHLH* gene increased proline level and Ca^2+^ concentration, decreased the accumulation of reactive oxygen species, and improved heat resistance.	[96]
MYB	*Camellia sinensis*	*CsMYB20*, *CsMYB21*	The transcription factors of *CsMYB20* and *CsMYB21* are upregulated and are involved in recognizing the conserved motifs in tea plants and inducing the expression of ABA synthesis reaction genes to resist stress.	[97]
WRKY	*Osmanthus fragrans*	*DlWRKY*	*DlWRKY2* reacted strongly to heat stress, while *DlWRKY36* and *DlWRKY46* mediated the expression of salicylic acid synthesis reaction gene to alleviate stress.	[98]
NAC	*Haloxylon ammodendron*	*HaNAC3*	*HaNAC3* can improve the tolerance of transgenic plants to high-temperature stress and participate in regulating the downstream genes and metabolic pathways of indolebutyric acid and abscisic acid.	[99]

## Data Availability

No new data were created or analyzed in this study. Data sharing is not applicable to this article.

## References

[1] Malunguja G.K., Devi A., Kilonzo M., Rubanza C.D. (2020). Climate change mitigation through carbon dioxide (CO_2_) sequestration in community reserved forests of northwest Tanzania. Arch. Agric. Environ. Sci..

[2] Hossain T., Jones D.S., Hartley D.S., Thompson D.N., Langholtz M., Davis M. (2022). Nth-plant scenario for forest resources and short rotation woody crops: Biorefineries and depots in the contiguous US. Appl. Energy..

[3] Sun Y.J., Zhou J., Guo J.S. (2021). Advances in the knowledge of adaptive mechanisms mediating abiotic stress responses in *Camellia sinensis*. Front. Biosci..

[4] Ruelland E., Zachowski A. (2010). How plants sense temperature. Environ. Exp. Bot..

[5] Choudhary S., Thakur S., Majeed A., Bhardwaj P. (2021). Adaptability of Rhododendrons in high altitude habitats. J. For. Res..

[6] Polgar C.A., Primack R.B. (2011). Leaf-out phenology of temperate woody plants: From trees to ecosystems. New Phytol..

[7] Wang Y., Lai L., Du H., Jiang L., Wang F., Zhang C., Zhuang P., Zheng Y. (2018). Phylogeny, habitat together with biological and ecological factors can influence germination of 36 subalpine *Rhododendron* species from the eastern Tibetan Plateau. Ecol. Evol..

[8] Guo C., Shen Y., Shi F. (2020). Effect of temperature, light, and storage time on the seed germination of *Pinus bungeana* Zucc. ex Endl.: The role of seed-covering layers and abscisic acid changes. Forests.

[9] Simoes I.M., Baptista J.O., Rosa T., de Mello T., de Araujo C.P., de Lima P., Dos Anjos B.B., de Oliveira J., Caldeira M., Otoni W.C. (2021). Heat stress affects the physiological and biochemical quality of *Dalbergia nigra* seeds in vitro. For. Sci..

[10] Kim D.H., Han S.H. (2018). Direct effects on seed germination of 17 tree species under elevated temperature and CO_2_ conditions. Open Life Sci..

[11] Santos M.M., Borges E.E.D.L., Ataíde G.D.M., Pires R.M.D.O., Rocha D.K. (2020). Enzyme activity in the micropylar region of *Melanoxylon brauna* Schott seeds during germination under heat stress conditions. J. Seed Sci..

[12] Barthélémy D., Caraglio Y. (2007). Plant architecture: A dynamic, multilevel and comprehensive approach to plant form, structure and ontogeny. Ann. Bot..

[13] Sehgal A., Sita K., Kumar J., Kumar S., Singh S., Siddique K., Nayyar H. (2017). Effects of drought, heat and their interaction on the growth, yield and photosynthetic function of lentil (*Lens culinaris* medikus) genotypes varying in heat and drought sensitivity. Front. Plant Sci..

[14] Wang J., Liu Y., Hu S., Xu J., Nian J., Cao X., Chen M., Cen J., Liu X., Zhang Z. (2022). Leaf tip rumpled 1 regulates leaf morphology and salt tolerance in rice. Int. J. Mol. Sci..

[15] Shafqat W., Jaskani M.J., Maqbool R., Chattha W.S., Ali Z., Naqvi S.A., Haider M.S., Khan I.A., Vincent C.I. (2021). Heat shock protein and aquaporin expression enhance water conserving behavior of citrus under water deficits and high temperature conditions. Environ. Exp. Bot..

[16] Burri S., Haeler E., Eugster W., Haeni M., Etzold S., Walthert L., Braun S., Zweifel R. (2019). How did Swiss forest trees respond to the hot summer 2015?. Die Erde.

[17] Xu H., Hannam K.D., MacDonald J.L., Ediger D. (2023). Field investigation into tree fates from recent apple tree decline: Abrupt hydraulic failure versus gradual hydraulic loss. Stresses.

[18] Jumrani K., Bhatia V.S. (2018). Impact of combined stress of high temperature and water deficit on growth and seed yield of soybean. Physiol. Mol. Biol. Plants.

[19] Seleiman M.F., Al-Suhaibani N., Ali N., Akmal M., Alotaibi M., Refay Y., Dindaroglu T., Abdul-Wajid H.H., Battaglia M.L. (2021). Drought stress impacts on plants and different approaches to alleviate its adverse effects. Plants.

[20] Lawas L.M.F., Zuther E., Jagadish S.K., Hincha D.K. (2018). Molecular mechanisms of combined heat and drought stress resilience in cereals. Curr. Opin. Plant Biol..

[21] Zhou Q., Jiang Z., Zhang X., Zhang T., Zhu H., Cui B., Li Y., Zhao F., Zhao Z. (2019). Leaf anatomy and ultrastructure in senescing ancient tree, *Platycladus orientalis* L. (Cupressaceae). Peer J..

[22] Li X.E., Zhao X., Tsujii Y., Ma Y.Q., Zhang R.Y., Qian C., Wang Z.X., Geng F.L., Jin S.X. (2022). Links between leaf anatomy and leaf mass per area of herbaceous species across slope aspects in an eastern Tibetan subalpine meadow. Ecol. Evol..

[23] Hong Y., Zhang L., Liu X., Aritsara A.N.A., Zeng X., Xing X., Lu Q., Wang K., Wang Y., Zhang Y. (2021). Tree ring anatomy indices of *Pinus tabuliformis* revealed the shifted dominant climate factor influencing potential hydraulic function in western Qinling Mountains. Dendrochronologia.

[24] Ruehr N.K., Grote R., Mayr S., Arneth A. (2019). Beyond the extreme: Recovery of carbon and water relations in woody plants following heat and drought stress. Tree Physiol..

[25] Stefi A.L., Papaioannou V., Nikou T., Halabalaki M., Vassilacopoulou D., Christodoulakis N.S. (2022). Heat and cold-stressed individuals of *Pistacia lentiscus* (Mastic Tree) do modify their secreting profile. Plants.

[26] Fajstavr M., Giagli K., Vavrcik H., Gryc V., Horacek P., Urban J. (2020). The cambial response of Scots pine trees to girdling and water stress. Iawa J..

[27] Zhao X., Li P., Liu X., Xu T., Zhang Y., Meng H., Xia T. (2022). High temperature increased lignin contents of poplar (*Populus* spp.) stem via inducing the synthesis caffeate and coniferaldehyde. Front. Genet..

[28] Karlova R., Boer D., Hayes S., Testerink C. (2021). Root plasticity under abiotic stress. Plant Physiol..

[29] El-Ramady H., Abdalla N., Elmahrouk M., Bayoumi Y., Elsakhawy T.A., Omara A.E., Shalaby T.A. (2021). Anatomical changes of cultivated plants under combined stress: An urgent need for investigation. Environ. Biodivers. Soil Secur..

[30] Singh R.K., Prasad A., Maurya J., Prasad M. (2022). Regulation of small RNA-mediated high temperature stress responses in crop plants. Plant Cell Rep..

[31] Xue J., Zeng P., Cui J., Zhang Y., Yang J., Zhu L., Hu H., Xu J. (2023). Physiological and gene expression changes of *Cryptomeria fortunei* Hooibrenk families under heat stress. Front. Plant Sci..

[32] Waadt R., Seller C.A., Hsu P., Takahashi Y., Munemasa S., Schroeder J.I. (2022). Plant hormone regulation of abiotic stress responses. Nat. Rev. Mol. Cell Biol..

[33] Grier C.G., Running S.W. (1977). Leaf area of mature northwestern coniferous forests: Relation to site water balance. Ecology.

[34] Andrasi N., Pettko-Szandtner A., Szabados L. (2021). Diversity of plant heat shock factors: Regulation, interactions, and functions. J. Exp. Bot..

[35] Li J.B., Zhao S., Yu X., Du W., Li H., Sun Y., Sun H., Ruan C.J. (2021). Role of *Xanthoceras sorbifolium* MYB44 in tolerance to combined drought and heat stress via modulation of stomatal closure and ROS homeostasis. Plant Physiol. Biochem..

[36] Knight H., Knight M.R. (2001). Abiotic stress signalling pathways: Specificity and cross-talk. Trends Plant Sci..

[37] Song Y., Chen Q., Ci D., Shao X., Zhang D. (2014). Effects of high temperature on photosynthesis and related gene expression in poplar. BMC Plant Biol..

[38] Liu C.L., Shen W.J., Yang C., Zeng L.Z., Gao C.J. (2018). Knowns and unknowns of plasma membrane protein degradation in plants. Plant Sci..

[39] Morais M.C., Ferreira H., Cabral J.A., Goncalves B., Rennenberg H. (2023). Differential tolerance of the woody invasive *Hakea sericea* to drought and terminal heat stress. Tree Physiol..

[40] You J., Chan Z. (2015). ROS regulation during abiotic stress responses in crop plants. Front. Plant Sci..

[41] Chen W., Yang W., Lo H., Yeh D. (2014). Physiology, anatomy, and cell membrane thermostability selection of leafy radish (*Raphanus sativus* var. oleiformis Pers.) with different tolerance under heat stress. Sci. Hortic..

[42] Schneider J.R., Caverzan A., Chavarria G. (2019). Water deficit stress, ROS involvement, and plant performance. Arch. Agron. Soil Sci..

[43] Shen H.F., Zhao B., Xu J.J., Liang W., Huang W.M., Li H.H. (2017). Effects of heat stress on changes in physiology and anatomy in two cultivars of *Rhododendron*. S. Afr. J. Bot..

[44] Rouina Y.B., Zouari M., Zouari N., Rouina B.B., Bouaziz M. (2020). Olive tree (*Olea europaea* L. cv. Zelmati) grown in hot desert climate: Physio-biochemical responses and olive oil quality. Sci. Hortic..

[45] Chao X., Yuqing T., Xincheng L., Huidong Y., Yuting W., Zhongdong H., Xinlong H., Buchun L., Jing S. (2022). Exogenous spermidine enhances the photosynthetic and antioxidant capacity of citrus seedlings under high temperature. Plant Signal. Behav..

[46] Li X., Wang Y., Zhang Y., Xiang J., Yang Z., Gu F., Wu M. (2022). Evaluating the physiological and biochemical responses of different mangrove species to upwelling. Front. Mar. Sci..

[47] Niu Y., Xiang Y. (2018). An overview of biomembrane functions in plant responses to high-temperature stress. Front. Plant Sci..

[48] Tiwari S., Patel A., Singh M., Prasad S.M., Tripathi D.K., Singh V.P., Chauhan D.K., Sharma S., Prasad S.M., Dubey N.K., Ramawat N. (2020). Regulation of temperature stress in plants. Plant Life under Changing Environment.

[49] Silva E.N., Ferreira-Silva S.L., de Vasconcelos Fontenele A., Ribeiro R.V., Viégas R.A., Silveira J.A.G. (2010). Photosynthetic changes and protective mechanisms against oxidative damage subjected to isolated and combined drought and heat stresses in *Jatropha curcas* plants. J. Plant Physiol..

[50] Verma S., Kumar N., Verma A., Singh H., Siddique K.H., Singh N.P. (2020). Novel approaches to mitigate heat stress impacts on crop growth and development. Plant Physiol. Rep..

[51] Dawood M.G. (2016). Influence of osmoregulators on plant tolerance to water stress. Sci. Agric..

[52] Sharma A., Shahzad B., Kumar V., Kohli S.K., Sidhu G., Bali A.S., Handa N., Kapoor D., Bhardwaj R., Zheng B. (2019). Phytohormones regulate accumulation of osmolytes under abiotic stress. Biomolecules.

[53] Huang H., Han Y., Hao J., Qin X., Liu C., Fan S. (2023). Exogenous spermidine modulates osmoregulatory substances and leaf stomata to alleviate the damage to lettuce seedlings caused by high temperature stress. J. Plant Growth Regul..

[54] Wang B.M., Chen J.J., Chen L.S., Wang X.N., Wang R., Ma L., Peng S.F., Luo J., Chen Y.Z. (2015). Combined drought and heat stress in *Camellia oleifera* cultivars: Leaf characteristics, soluble sugar and protein contents, and *Rubisco* gene expression. Trees-Struct. Funct..

[55] Marias D.E., Meinzer F.C., Woodruff D.R., McCulloh K.A. (2017). Thermotolerance and heat stress responses of Douglas-fir and ponderosa pine seedling populations from contrasting climates. Tree Physiol..

[56] Saksena H.B., Sharma M., Singh D., Laxmi A. (2020). The versatile role of glucose signalling in regulating growth, development and stress responses in plants. J. Plant Biochem. Biotechnol..

[57] Salvi P., Agarrwal R., Kajal, Gandass N., Manna M., Kaur H., Deshmukh R. (2022). Sugar transporters and their molecular tradeoffs during abiotic stress responses in plants. Physiol. Plant..

[58] Iqbal N., Umar S., Khan N.A., Corpas F.J. (2021). Crosstalk between abscisic acid and nitric oxide under heat stress: Exploring new vantage points. Plant Cell Rep..

[59] Liu J., Wang Y.S. (2020). Proline metabolism and molecular cloning of AmP5CS in the mangrove *Avicennia marina* under heat stress. Ecotoxicology.

[60] Chang B., Ma K., Lu Z., Lu J., Cui J., Wang L., Jin B. (2020). Physiological, transcriptomic, and metabolic responses of *Ginkgo biloba* L. to drought, salt, and heat stresses. Biomolecules.

[61] Nadarajah K.K. (2020). ROS homeostasis in abiotic stress tolerance in plants. Int. J. Mol. Sci..

[62] Zandalinas S.I., Mittler R., Balfagón D., Arbona V., Gómez Cadenas A. (2018). Plant adaptations to the combination of drought and high temperatures. Physiol. Plant..

[63] Li Y., Liang W., Zhao B. (2020). Physiological and microstructural responses of two *Rhododendron* cultivars to high temperature and low light. Hortic. Environ. Biotechnol..

[64] Rascio A., Altamura G., Pecorella I., Goglia L., Sorrentino G. (2023). Physiological mechanisms preventing plant wilting under heat stress: A case study on a wheat (*Triticum durum* desf.) bound water-mutant. Environ. Exp. Bot..

[65] Rajput V.D., Harish, Singh R.K., Verma K.K., Sharma L., Quiroz-Figueroa F.R., Meena M., Gour V.S., Minkina T., Sushkova S. (2021). Recent developments in enzymatic antioxidant defence mechanism in plants with special reference to abiotic stress. Biology.

[66] Dvořák P., Krasylenko Y., Zeiner A., Šamaj J., Takáč T. (2021). Signaling toward reactive oxygen species-scavenging enzymes in plants. Front. Plant Sci..

[67] Yamane K., Nishikawa M., Hirooka Y., Narita Y., Kobayashi T., Kakiuchi M., Iwai K., Iijima M. (2022). Temperature tolerance threshold and mechanism of oxidative damage in the leaf of Coffea arabica ‘Typica’ under heat stress. Plant Prod. Sci..

[68] Liu L., Lin W., Zhang L., Tang X., Liu Y., Lan S., Wang S., Zhou Y., Chen X., Wang L. (2022). Changes and correlation between physiological characteristics of *Rhododendron simsii* and soil microbial communities under heat stress. Front. Plant Sci..

[69] Karabulut G., Feng H., Yemiş O. (2022). Physicochemical and antioxidant properties of industrial hemp seed protein isolate treated by high-intensity ultrasound. Plant Food Hum. Nutr..

[70] Han X., Yao F., Xue T., Wang Z., Wang Y., Cao X., Hui M., Wu D., Li Y., Wang H. (2022). Sprayed Biodegradable Liquid Film Improved the Freezing Tolerance of cv. Cabernet sauvignon by Up-Regulating Soluble Protein and Carbohydrate Levels and Alleviating Oxidative Damage. Front. Plant Sci..

[71] Jiang J.F., Liu X.N., Liu C.H., Liu G.T., Li S.H., Wang L.J. (2017). Integrating omics and alternative splicing reveals insights into grape response to high temperature. Plant Physiol..

[72] Siddiqui H., Hayat S., Bajguz A. (2018). Regulation of photosynthesis by brassinosteroids in plants. Acta Physiol. Plant..

[73] Zahra N., Hafeez M.B., Ghaffar A., Kausar A., Al Zeidi M., Siddique K.H., Farooq M. (2023). Plant photosynthesis under heat stress: Effects and management. Environ. Exp. Bot..

[74] Perez-Oliver M.A., Haro J.G., Pavlovic I., Novak O., Segura J., Sales E., Arrillaga I. (2021). Priming maritime pine megagametophytes during somatic embryogenesis improved plant adaptation to heat stress. Plants.

[75] Hu S., Ding Y., Zhu C. (2020). Sensitivity and responses of chloroplasts to heat stress in plants. Front. Plant Sci..

[76] Chaudhry S., Sidhu G. (2022). Climate change regulated abiotic stress mechanisms in plants: A comprehensive review. Plant Cell Rep..

[77] Dias M.C., Correia S., Serôdio J., Silva A.M.S., Freitas H., Santos C. (2018). Chlorophyll fluorescence and oxidative stress endpoints to discriminate olive cultivars tolerance to drought and heat episodes. Sci. Hortic..

[78] Twala T.C., Witkowski E.F., Fisher J.T. (2022). The effects of heat and drought stress on the ecophysiological responses and growth of *Afrocarpus falcatus* and *Podocarpus henkelii* seedlings. S. Afr. J. Bot..

[79] Zhang K., Chen B.H., Hao Y., Yang R., Wang Y.A. (2018). Effects of short-term heat stress on PSII and subsequent recovery for senescent leaves of *Vitis vinifera* L. cv. Red Globe. J. Integr. Agric..

[80] Dewhirst R.A., Handakumbura P., Clendinen C.S., Arm E., Tate K., Wang W., Washton N.M., Young R.P., Mortimer J.C., McDowell N.G. (2021). High temperature acclimation of leaf gas exchange, photochemistry, and metabolomic profiles in *Populus trichocarpa*. ACS Earth Space Chem..

[81] Haider S., Iqbal J., Naseer S., Shaukat M., Abbasi B.A., Yaseen T., Zahra S.A., Mahmood T. (2022). Unfolding molecular switches in plant heat stress resistance: A comprehensive review. Plant Cell Rep..

[82] Guo M., Liu J.H., Ma X., Luo D.X., Gong Z.H., Lu M.H. (2016). The plant heat stress transcription factors (HSFs): Structure, regulation, and function in response to abiotic stresses. Front. Plant Sci..

[83] Tian F., Hu X.L., Yao T., Yang X., Chen J.G., Lu M.Z., Zhang J. (2021). Recent advances in the roles of HSFs and HSPs in heat stress response in woody plants. Front. Plant Sci..

[84] Driedonks N., Xu J., Peters J.L., Park S., Rieu I. (2015). Multi-level interactions between heat shock factors, heat shock proteins, and the redox system regulate acclimation to heat. Front. Plant Sci..

[85] Du W., Ruan C.J., Li J.B., Li H., Ding J., Zhao S.Y., Jiang X. (2021). Quantitative proteomic analysis of *Xanthoceras sorbifolium* Bunge seedlings in response to drought and heat stress. Plant Physiol. Biochem..

[86] Elfving N., Davoine C., Benlloch R., Blomberg J., Brännström K., Müller D., Nilsson A., Ulfstedt M., Ronne H., Wingsle G. (2011). The *Arabidopsis thaliana* Med25 mediator subunit integrates environmental cues to control plant development. Proc. Natl. Acad. Sci. USA.

[87] Kiranmai K., Lokanadha R.G., Pandurangaiah M., Nareshkumar A., Amaranatha R.V., Lokesh U., Venkatesh B., Anthony J.A., Sudhakar C. (2018). A novel WRKY transcription factor, MuWRKY3 (*Macrotyloma uniflorum* Lam. Verdc.) enhances drought stress tolerance in transgenic groundnut (*Arachis hypogaea* L.). Plants Front. Plant Sci..

[88] Xu P., Guo Q., Pang X., Zhang P., Kong D., Liu J. (2020). New insights into evolution of plant heat shock factors (Hsfs) and expression analysis of tea genes in response to abiotic stresses. Plants.

[89] Yuan T., Liang J., Dai J., Zhou X.R., Liao W., Guo M., Aslam M., Li S., Cao G., Cao S. (2022). Genome-Wide identification of eucalyptus heat shock transcription factor family and their transcriptional analysis under salt and temperature stresses. Int. J. Mol. Sci..

[90] Liu X., Meng P., Yang G., Zhang M., Peng S., Zhai M.Z. (2020). Genome-wide identification and transcript profiles of walnut heat stress transcription factor involved in abiotic stress. BMC Genom..

[91] Lin Q., Jiang Q., Lin J.Y., Wang D.L., Li S.J., Liu C.R., Sun C.D., Chen K.S. (2015). Heat shock transcription factors expression during fruit development and under hot air stress in Ponkan (*Citrus reticulata* Blanco cv. Ponkan) fruit. Gene.

[92] Wan X., Yang J., Guo C., Bao M., Zhang J. (2019). Genome-wide identification and classification of the *Hsf* and *sHsp* gene families in *Prunus mume*, and transcriptional analysis under heat stress. Peer J..

[93] Liu G.T., Chai F.M., Wang Y., Jiang J.Z., Duan W., Wang Y.T., Wang F.F., Li S.H., Wang L.J. (2018). Genome-wide identification and classification of HSF family in grape, and their transcriptional analysis under heat acclimation and heat stress. Hortic. Plant J..

[94] Panzade K.P., Kale S.S., Kapale V., Chavan N.R. (2021). Genome-Wide analysis of heat shock transcription factors in *Ziziphus jujuba* identifies potential candidates for crop improvement under abiotic stress. Appl. Biochem. Biotechnol..

[95] Li H., Yang Y., Liu M., Zhu Y., Wang H., Feng C., Niu M., Liu C., Yin W., Xia X. (2022). The in vivo performance of a heat shock transcription factor from *Populus euphratica*, PeHSFA2, promises a prospective strategy to alleviate heat stress damage in poplar. Environ. Exp. Bot..

[96] Ji X.Y., Nie X.G., Liu Y.J., Zheng L., Zhao H.M., Zhang B., Huo L., Wang Y.C. (2016). A *bHLH* gene from *Tamarix hispida* improves abiotic stress tolerance by enhancing osmotic potential and decreasing reactive oxygen species accumulation. Tree Physiol..

[97] Wang W.L., Cui X., Wang Y.X., Liu Z.W., Zhuang J. (2018). Members of R2R3-type MYB transcription factors from subgroups 20 and 22 are involved in abiotic stress response in tea plants. Biotechnol. Biotechnol. Equip..

[98] Jue D., Sang X., Liu L., Shu B., Wang Y., Liu C., Xie J., Shi S. (2018). Identification of WRKY gene family from *Dimocarpus longan* and its expression analysis during flower induction and abiotic stress responses. Int. J. Mol. Sci..

[99] Liu X., Zong X., Wu X., Liu H., Han J., Yao Z., Ren Y., Ma L., Wang B., Zhang H. (2022). Ectopic expression of NAC transcription factor HaNAC3 from *Haloxylon ammodendron* increased abiotic stress resistance in tobacco. Planta.

[100] Sangster T.A., Queitsch C. (2005). The HSP90 chaperone complex, an emerging force in plant development and phenotypic plasticity. Curr. Opin. Plant Biol..

[101] Liu J., Feng L., Li J., He Z. (2015). Genetic and epigenetic control of plant heat responses. Front. Plant Sci..

[102] Alhassan D.A. (2023). Advances in Differentially Methylated Region Detection and Cure Survival Models.

[103] Sun M., Yang Z., Liu L., Duan L. (2022). DNA methylation in plant responses and adaption to abiotic stresses. Int. J. Mol. Sci..

[104] Correia B., Valledor L., Meijon M., Rodriguez J.L., Dias M.C., Santos C., Canal M.J., Rodriguez R., Pinto G. (2013). Is the interplay between epigenetic markers related to the acclimation of cork oak plants to high temperatures?. PLoS ONE.

[105] Ci D., Song Y., Tian M., Zhang D. (2015). Methylation of miRNA genes in the response to temperature stress in *Populus simonii*. Front. Plant Sci..

[106] Hossain M.S., Kawakatsu T., Kim K.D., Zhang N., Nguyen C.T., Khan S.M., Batek J.M., Joshi T., Schmutz J., Grimwood J. (2017). Divergent cytosine DNA methylation patterns in single-cell, soybean root hairs. New Phytol..

[107] Li J., Huang Q., Sun M., Zhang T., Li H., Chen B., Xu K., Gao G., Li F., Yan G. (2016). Global DNA methylation variations after short-term heat shock treatment in cultured microspores of brassica napus cv. Topas. Sci. Rep..

[108] Malabarba J., Windels D., Xu W., Verdier J. (2021). Regulation of DNA (de)methylation positively impacts seed germination during seed development under heat stress. Genes.

[109] Liu J., He Z. (2020). Small DNA methylation, big player in plant abiotic stress responses and memory. Front. Plant Sci..

[110] Cui X., Zheng Y., Lu Y., Issakidis-Bourguet E., Zhou D. (2021). Metabolic control of histone demethylase activity involved in plant response to high temperature. Plant Physiol..

[111] Liu C., Lu F., Cui X., Cao X. (2010). Histone methylation in higher plants. Annu. Rev. Plant Biol..

[112] Li B., Yang C., An B., Wang H., Albaqami M., Abou Elwafa S.F., Xu L., Xu Y. (2022). Comparative transcriptomic and epigenetic analyses reveal conserved and divergent regulatory pathways in barley response to temperature stresses. Physiol. Plant..

[113] Liu G., Jiang J., Liu X., Jiang J., Sun L., Duan W., Li R., Wang Y., Lecourieux D., Liu C. (2019). New insights into the heat responses of grape leaves via combined phosphoproteomic and acetylproteomic analyses. Hortic. Res..

